# Neurotrophin/Trk receptor signaling mediates C/EBPα, -β and NeuroD recruitment to immediate-early gene promoters in neuronal cells and requires C/EBPs to induce immediate-early gene transcription

**DOI:** 10.1186/1749-8104-2-4

**Published:** 2007-01-25

**Authors:** Anna Maria Calella, Claus Nerlov, Rodolphe G Lopez, Carla Sciarretta, Oliver von Bohlen und Halbach, Oksana Bereshchenko, Liliana Minichiello

**Affiliations:** 1 European Molecular Biology Laboratory, Mouse Biology Unit, via Ramarini, 00016 Monterotondo, Italy; 2 Interdisciplinary Center for Neurosciences (IZN), Department of Neuroanatomy, University of Heidelberg, Im Neuenheimer Feld, 69120 Heidelberg, Germany; 3 University Hospital Zurich, Institute for Neuropathology, Schmelzbergstrasse, 8091 Zurich, Switzerland

## Abstract

**Background:**

Extracellular signaling through receptors for neurotrophins mediates diverse neuronal functions, including survival, migration and differentiation in the central nervous system, but the transcriptional targets and regulators that mediate these diverse neurotrophin functions are not well understood.

**Results:**

We have identified the immediate-early (IE) genes *Fos*, *Egr1 *and *Egr2 *as transcriptional targets of brain derived neurotrophic factor (BDNF)/TrkB signaling in primary cortical neurons, and show that the *Fos *serum response element area responds to BDNF/TrkB in a manner dependent on a combined C/EBP-Ebox element. The *Egr1 *and *Egr2 *promoters contain homologous regulatory elements. We found that C/EBPα/β and NeuroD formed complexes *in vitro *and *in vivo*, and were recruited to all three homologous promoter regions. C/EBPα and NeuroD co-operatively activated the *Fos *promoter in transfection assays. Genetic depletion of Trk receptors led to impaired recruitment of C/EBPs and NeuroD *in vivo*, and elimination of *Cebpa *and *Cebpb *alleles reduced BDNF induction of *Fos*, *Egr1 *and *Egr2 *in primary neurons. Finally, defective differentiation of cortical dendrites, as measured by MAP2 staining, was observed in both compound *Cebp *and *Ntrk *knockout mice.

**Conclusion:**

We here identify three IE genes as targets for BDNF/TrkB signaling, show that C/EBPα and -β are recruited along with NeuroD to target promoters, and that C/EBPs are essential mediators of Trk signaling in cortical neurons. We show also that C/EBPs and Trks are required for cortical dendrite differentiation, consistent with Trks regulating dendritic differentiation via a C/EBP-dependent mechanism. Finally, this study indicates that BDNF induction of IE genes important for neuronal function depends on transcription factors (C/EBP, NeuroD) up-regulated during neuronal development, thereby coupling the functional competence of the neuronal cells to their differentiation.

## Background

The influence of growth factors, as well as transcription factors, on cell fate determination and differentiation is well established. Brain derived neurotrophic factor (BDNF) affects neuronal cortical development by signaling through its cognate tyrosine kinase receptor, TrkB. Together they regulate many cellular functions during and after the development of the nervous system [1-4]. In particular, we have reported that TrkB, via the activation of the Ras/Mitogen activated protein kinase (MAPK) and Phosphatidylinositol-3 kinase/cellular homolog of the viral oncogene v-Akt (PI3K/AKT) pathways, regulates functions throughout the formation of the cerebral cortex, including neuronal dendritic differentiation [5]. While several transcription factors have been found to respond to activation of TrkB and other Trk family receptors, it is not well understood how Trk signaling interacts with the transcription factors regulating neuronal differentiation to activate specific target genes required during the different phases of neuronal development and, subsequently, in neuronal function.

The neurogenic basic helix-loop-helix (bHLH) transcription factors have been shown to be important intrinsic regulators of neural fate determination and differentiation. There are at least two categories of bHLH transcription factors that mediate neurogenesis: proneural bHLHs (Neurogenins and Mash family proteins), which are involved in initiating neurogenesis, and neuronal differentiation factors (members of the NeuroD/Nex family), which are important for terminal differentiation. Studies in mice lacking NeuroD/Nex family members indicate that they are required not only for the survival of early differentiating granule neurons in the hippocampus and cerebellum, but also for their terminal differentiation [6,7]. Specifically, NeuroD selectively promotes dendrite, but not axonal, morphogenesis in granule neurons through its phosphorylation at distinct sites by CamKII [8]. These factors form heterodimers with ubiquitously expressed bHLH proteins, such as E12 and E47, and activate gene expression programs through interaction, via their basic domain, with DNA sequences containing the core hexanucleotide motif CANNTG, known as the Ebox [9,10]. It is still unknown which extracellular signals or intracellular mechanisms are involved in the regulation of bHLH factor function.

The CCAAT/enhancer binding protein (C/EBP) family of transcription factors couples growth factor signal transduction with numerous cellular responses, including cellular differentiation in a variety of non-neuronal tissues [11,12]. The expression of C/EBPs has also been found to change during a number of physiological and pathophysiological conditions in response to extracellular signals, such as hormones, mitogens, and cytokines [12]. Recently, a Mitogen and extracellular signal regulated kinase (MEK)-C/EBP signaling cascade has been implicated in the commitment of progenitor cells toward a neuronal fate *in vitro *through the inhibition of either MEK or C/EBPs using dominant negative forms [13]. Paquin *et al*. [14], using *in utero *electroporation to manipulate cortical precursors, have suggested that MEK-mediated phosphorylation of C/EBPs is essential for neuronal fate determination. Thus far, however, no genetic model has demonstrated an effect for MEK1/2 or C/EBPs on neuronal lineage commitment or differentiation.

We here identify *Fos*, *Egr1 *and *Egr2 *as genes that are regulated by BDNF/TrkB in a C/EBP-dependent manner. We identify a novel complex between C/EBPα/β and NeuroD, and find that *in vivo*, these factors are recruited to the *Fos*, *Egr1 *and *Egr2 *promoters in a Trk-dependent manner. Genetic C/EBP depletion leads to a cortical dendritic neuronal differentiation defect, reminiscent of that seen in the absence of multiple TrkB/C alleles, consistent with the idea that Trk signaling through C/EBP-NeuroD complexes mediates terminal cortical neuronal differentiation *in vivo*.

## Results

### BDNF induces *Egr1*, *Egr2 *and *Fos *expression in primary cortical neurons

**Figure 1:**
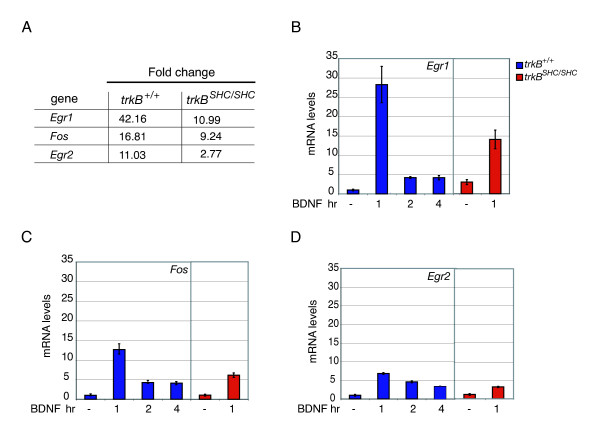
IE gene expression upon BDNF stimulation of primary cortical neurons. **(a) **Comparison of selected group of genes between *trkB*^+/+ ^and *trkB*^*SHC*/*SHC *^mutant mice. The mRNA for the Affymetrix analysis was obtained from E15.5 primary cortical neurons with +/+ or TrkB^SHC ^receptors that were either left unstimulated or stimulated with 50 ng/ml of BDNF for 1 hour after 1DIV. The fold change was averaged from three independent experiments. **(b-d) **qRT-PCR analysis of the selected BDNF regulated genes. Total mRNA obtained from *trkB*^+/+ ^or *trkB*^*SHC*/*SHC *^primary cortical neurons, either left untreated or treated with 50 ng/ml of BDNF at different time points, were analyzed by qRT-PCR using primers specific for *Egr1 *(b), *Fos *(c), and *Egr2 *(d). After normalization to GAPDH, mRNA levels were expressed relative to the levels obtained from wild-type unstimulated cortical neurons, which were given the arbitrary value of 1. Average values and standard errors were obtained from three independent animals.

To identify genes regulated by BDNF signaling through TrkB in neuronal cells, primary cortical neurons derived from E15.5 embryonic forebrain (when neurogenesis is at its peak) were stimulated with BDNF and their gene expression compared to that of untreated control cultures by microarray analysis. Among the most highly regulated genes was a group encoding immediate-early (IE) transcription factors that consisted of *Egr1*, *Egr2 *and *Fos*. For all three it was found that BDNF induction was reduced when the binding site for the Shc adaptor on the TrkB receptor was mutated (for this mouse strain see [5,15], and Materials and methods), consistent with a common regulatory mechanism underlying their upregulation (Figure 1a). The Shc site-dependent induction of these genes was confirmed by quantitative real time PCR (qRT-PCR) (Figure 1b–d).

### A combined C/EBP site-E-box mediates *Fos *induction by BDNF

**Figure 2:**
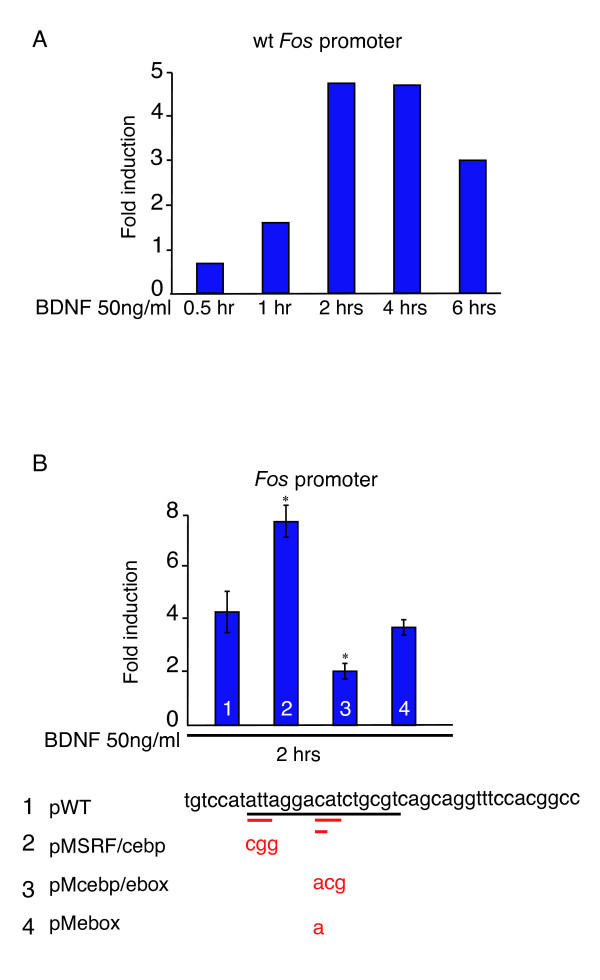
A combined C/EBP binding site-E-box mediates *Fos *induction by BDNF/TrkB signaling in primary cortical neurons. **(a) **Time course of luciferase activity based upon BDNF stimulation (50 ng/ml) of E15.5 wild-type (wt) cortical neurons transfected with a human *Fos *(*hFos*) promoter luciferase reporter plasmid (the proximal region of the *Fos *promoter in mouse and human is identical; see supplemental Figure 2a in Additional data file 1) and a pRSV-β-gal plasmid. Maximum luciferase activity was found between 2 and 4 hours of BDNF stimulation. **(b) **The combined C/EBP and Ebox sites are fundamental for the activation of the *Fos *promoter downstream of BDNF/TrkB. E15.5 wild-type primary cortical neurons were transfected with either wild-type or mutated *Fos *promoter luciferase reporter plasmids and pRSV-β-gal, and stimulated with 50 ng/ml of BDNF for 2 hours. Data shown are the average of three independent experiments, and results are indicated as the mean ± standard error. Cells transfected with a *Fos *promoter mutated in both the C/EBP and the Ebox binding sites (pMcebp/ebox) showed a significant decrease in luciferase activity (*p < 0.005 relative to pWT). Instead, cortical neurons transfected with a plasmid mutated in the SRE and C/EBP binding site (pMSRF/cebp) revealed a significant increment in the activation of the promoter (*p < 0.005 relative to pWT). No significant difference in luciferase activity was observed between the wild-type *Fos *promoter (pWT) and the *Fos *promoter mutated at the Ebox alone (pMebox).

**Figure 3:**
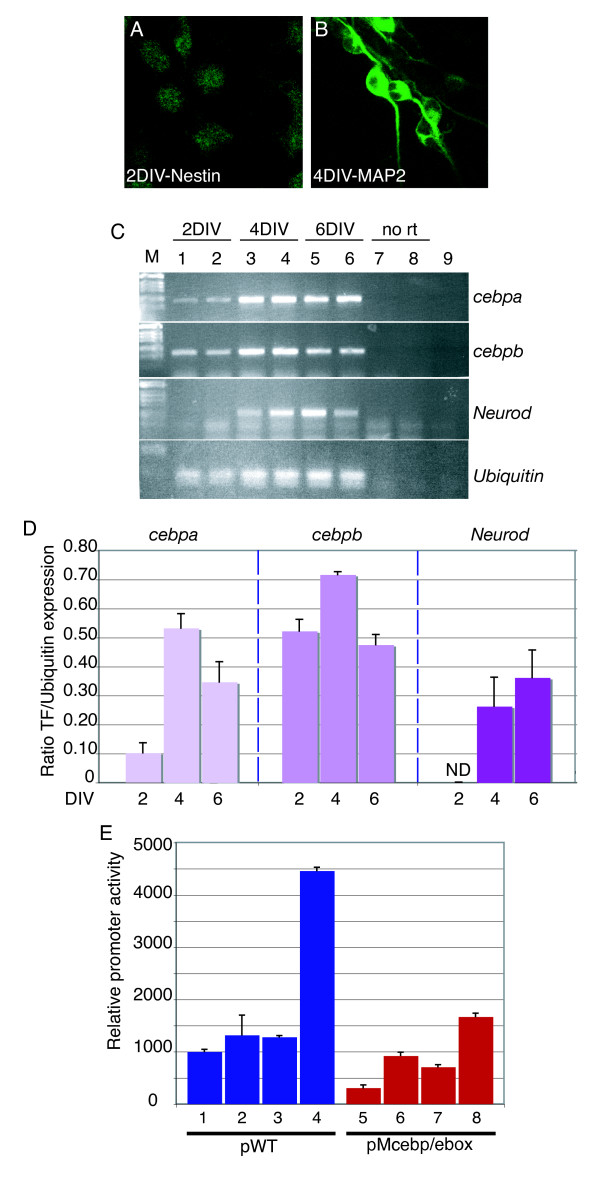
C/EBPα/β and NeuroD transcription factors are co-expressed in neurons and cooperate to activate the *Fos *promoter. **(a,b) **Immunocytochemical analysis of cortical neuron progenitors cultured at E12.5, and stained with Nestin antibodies after 2DIV (a) or antibodies against the differentiation marker MAP2 after 4DIV (b). **(c) **RT-PCR analysis of *Cebpa/b *and *Neurod *in cortical progenitors. Primary cortical neuron progenitors from two different preparations were cultured at E12.5 and harvested for total mRNA after 2DIV (lanes 1 and 2), 4DIV (lanes 3 and 4), or 6DIV (lanes 5 and 6). *Ubiquitin *was used to control the levels of mRNA. Lanes 7 and 8 are mRNA samples without reverse transcriptase (RT). Lane 9, no template. **(d) **RT-PCR shown in (c) was quantified by densitometric analysis and is shown as the ratio of transcription factor (TF) to *ubiquitin *expression level. **(e) **Cooperation between C/EBPα and NeuroD transcription factors in the activation of the *Fos *promoter. NIH3T3 cells were transfected with either a wild-type *Fos *reporter plasmid (pWT, blue bars) or a reporter plasmid in which both the C/EBP and the Ebox binding sites were mutated (pMcebp/ebox, red bars), in the absence (lanes 1 and 5) or presence of a plasmid containing either C/EBPα (lanes 2 and 6), NeuroD (lanes 3 and 7) or a combination of both plasmids C/EBPα and NeuroD (lanes 4 and 8). Co-transfection of C/EBPα and NeuroD significantly increased the activation of the wild-type *Fos *promoter (lane 4) compared to C/EBPα or NeuroD alone (p < 0.0001 in both cases). This cooperation was significantly reduced (p < 0.0001) upon mutation of the C/EBP and Ebox binding sites (lane 8). Luciferase activity was normalised to β-gal activity. The relative luciferase activity represents the mean value of at least four independent experiments.

In order to identify the transcriptional mechanism by which BDNF induces IE gene transcription we initially established that a reporter construct carrying 700 base-pairs (bp) of the *Fos *promoter was induced by BDNF when transiently transfected into cultures of primary cortical neurons (Figure 2a). We next analyzed the role of the area of the *Fos *promoter surrounding the serum response element (SRE), as this is the most highly conserved part of the proximal *Fos *promoter. The SRE area contains overlapping binding sites for serum response factor (SRF), C/EBP, E-proteins and activating protein-1 (AP-1)/activating transcription factor (ATF). By transient transfection analysis, the simultaneous mutation of the Ebox and C/EBP site (see supplementary Figure 1 in Additional data file 1 and Supplemental materials and methods in Additional data file 2 for analysis) was found to significantly decrease promoter induction by BDNF (Figure 2b). We determined that both *Cebpa *and *Cebpb*, as well as *Neurod *(encoding the Ebox protein Beta2/NeuroD), were upregulated in neuroblasts cultured under neural differentiation conditions (Figure 3a–d), and that C/EBPα and NeuroD cooperated in *Fos *promoter activation, in a manner dependent on the presence of their cognate binding sites, in a heterologous cell line not expressing these proteins (Figure 3e). Here it should be noticed that other C/EBPα and NeuroD sites might be present in the promoter or in the vector backbone, providing residual activation after mutation of the identified C/EBPα and NeuroD sites.

### C/EBPs and NeuroD are recruited to IE gene promoters *in vivo*

**Figure 4:**
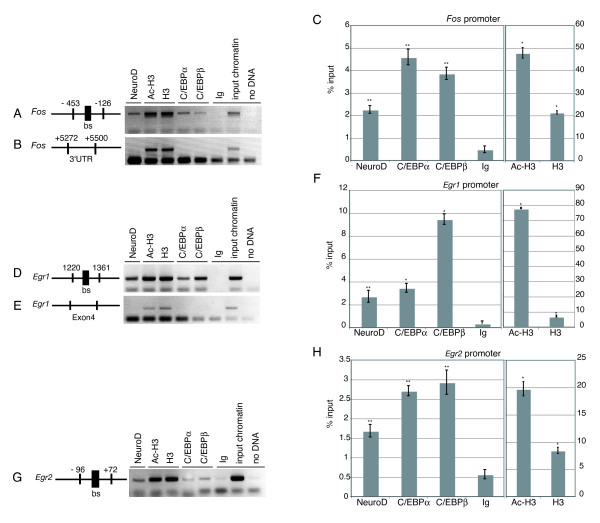
C/EBPα/β and NeuroD are bound to the *Fos*, *Egr1*, and *Egr2 *promoters *in vivo*. **(a,d,g) ***In vivo *detection of *Fos*, *Egr1*, or *Egr2 *promoter occupancy by C/EBPα/β and NeuroD using ChIP analysis. The schemes illustrate the *Fos*, *Egr1*, and *Egr2 *promoter regions amplified by PCR in their 5' UTR (nucleotides -453 to -126, 1220 to 1361, and -96 to 72, respectively). The large black boxes indicate the predicted DNA binding sites (bs) for C/EBP and NeuroD. The small boxes indicate the position of the primers used for PCR amplification. ChIP assays were performed using E16.5 wild-type forebrain lysates and antibodies against NeuroD, C/EBPα and β, and demonstrated that these proteins are recruited to the three promoters. Input corresponds to PCR reactions containing 0.5% of the total amount of chromatin used in IP reactions. **(b,e) **To confirm the specificity of the results, IPs from each antibody were also analyzed by PCR with primers specific for a region of the *Fos *gene located in the 3' UTR, or for a region of the *Egr1 *gene located in exon 4 as indicated in the schemes. **(c,f,h) **qPCR analysis was used to quantify the relative amounts of DNA obtained by ChIP for the *Fos *(c), *Egr1 *(f), and *Egr2 *(h) promoters. DNA quantities (expressed as percentages of input) were compared for specific IPs versus Ig IP samples (see Materials and methods). The corresponding *p *values are indicated: *p < 0.002, **p < 0.02 for *Egr1*; *p < 0.0001, **p < 0.005 for *Fos*; *p < 0.0001, **p < 0.002 for *Egr2*. Error bars represent the standard error based on three independent experiments.

Sequence alignment identified homologies to the *Fos *SRE area in the *Egr1 *and *Egr2 *promoters (supplemental Figure 2 in Additional data file 1). To understand if these homologous promoter regions bound C/EBPs and NeuroD in neuronal cells *in vivo*, chromatin immunoprecipitation (ChIP) analysis was performed on embryonic forebrains. This showed that C/EBPα, C/EBPβ and NeuroD were all present on the three homologous promoter regions (Figure 4a, c, d, f–h). As a control for specificity of the ChIP, a similar analysis was performed on two unrelated sequences derived from the *Fos *3' untranslated region (UTR) and *Egr1 *exon 4; these regions showed no detectable binding of C/EBPs or NeuroD, whereas antibodies against histone 3 (H3) and acetyl-H3 (Ac-H3) were able to precipitate these amplicons, confirming the quality of the input chromatin (Figure 4b, e).

### C/EBPs mediate BDNF induction of IE genes in primary cortical neurons

**Figure 5:**
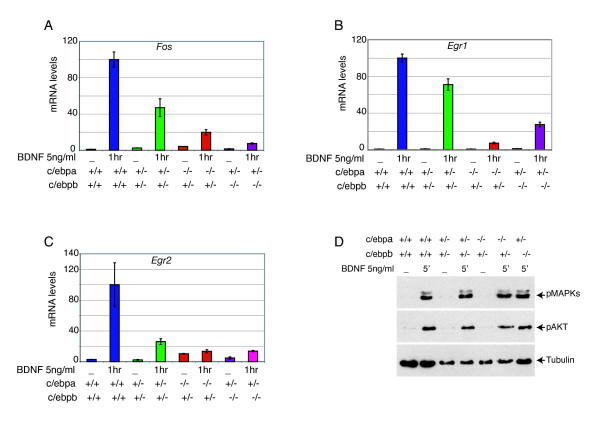
BDNF dependent expression of *Fos*, *Egr1 *and *Egr2 *is affected in mice with compound *Cebpa/b *null mutant alleles. **(a-c) **qRT-PCR analysis was performed on mRNA obtained from E13.5 primary cortical neurons of wild-type and different genotypes for *Cebpa/b *null mutants as indicated. After 3DIV, the neurons were either left unstimulated or stimulated with 5 ng/ml of BDNF for 1 hour. The mRNA levels are relative, after normalization to *gapdh*, considering the levels for the stimulated wild-type cortical neurons as 100. Error bars represent the standard error obtained from three animals for each genotype. *P *< 0.0001 for all induced samples of mutants compared to wild type. **(d) **BDNF/TrkB dependent signaling in primary cortical neurons with mutant *Cebpa/b*. E13.5 cortical neurons derived from wild-type or different genotypes for *Cebpa/b *null mutants after 3DIV were either left unstimulated or stimulated with 5 ng/ml of BDNF for 5 minutes. Cell lysates were subjected to SDS-PAGE followed by immunoblotting with an antibody against the phosphorylated forms of ERK (p42/p44). The blots were re-probed with an anti-phospho-AKT antibody, and a second time with an antibody against α-tubulin to control for the amount of protein loaded.

The above analysis showed that the *Fos*, *Egr1 *and *Egr2 *promoters contain homologous regulatory sequences, and that all three recruit C/EBPα, -β and NeuroD. To determine whether C/EBPs play a direct role in the BDNF regulation of these genes, we cultured primary cortical neurons from mice carrying two or three null alleles of the *Cebpa *and *Cebpb *genes (Figure 5a–c); inactivation of all four *Cebpa *and *Cebpb *alleles leads to embryonic lethality at E10.5 [16]. This analysis showed that a significant decrease in expression after BDNF stimulation was observed already in *Cebpa*^+/-^*Cebpb*^+/- ^cells, whereas further loss of an additional allele (*Cebpa*^+/-^*Cebpb*^-/- ^or *Cebpa*^-/-^*Cebpb*^+/- ^cells) led to a further reduction, resulting in loss of 70% to 90% of the BDNF-induced level of gene expression. This was not due to loss of BDNF/TrkB signaling, as BDNF-mediated induction of the MAPK pathway (as measured by p42/p44 MAPK phosphorylation) and the PI3K/AKT pathway (as measured by AKT phosphorylation) were unaffected by loss of *Cebpa *and *Cebpb *alleles (Figure 5d).

### Trk signaling regulates promoter recruitment

**Figure 6:**
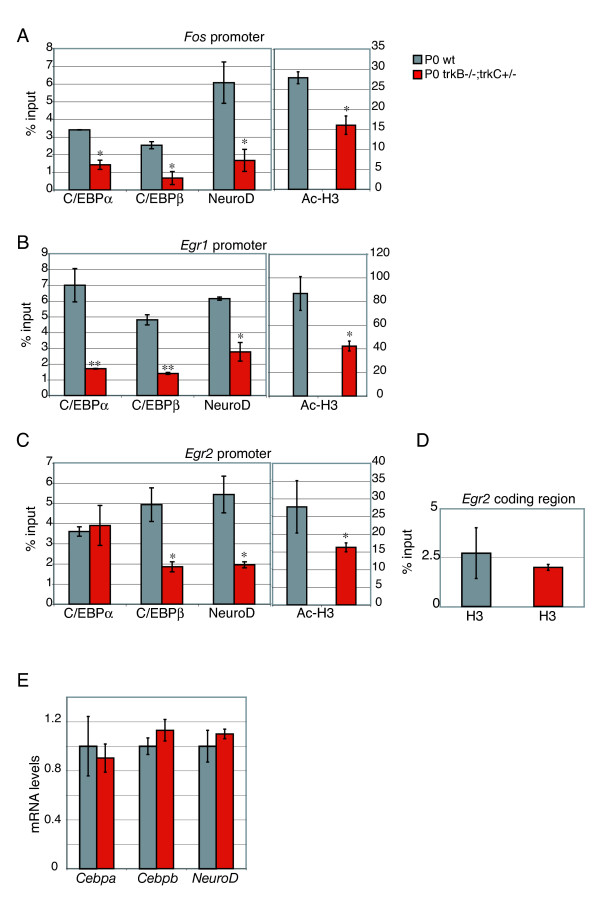
The presence of CEBPα/β and NeuroD on the *Fos*, *Egr1 *and *Egr2 *promoters is dependent on Trk receptor signaling. **(a-c) ***In vivo *detection of *Fos*, *Egr1 *and *Egr2 *promoter occupancy by C/EBPs and NeuroD in the absence of multiple *trkB/C *alleles. ChIP analysis was performed three times on a pool of four newborn forebrain lysates from wild-type (wt) and *trkB*^-/-^*;trkC*^+/- ^mice using antibodies specific for C/EBPα, C/EBPβ, NeuroD, and acetylated-H3 (Ac-H3). Following ChIP analysis the samples were subjected to qPCR, and the DNA quantification was calculated as percentage of the input. In all cases, no PCR product was detected in the absence of DNA. The *p *value refers to the difference between wild-type and mutant animals. **(d,e) **Control experiments. **(d) **H3 ChIP analysis and qPCR were performed with primer pairs located in the *Egr2*-coding region. A similar amount of PCR product was detected in wild-type and mutant animals. **(e) ***Cebpa*, *Cebpb *and *Neurod *mRNA levels were not affected in the absence of Trk signaling. qRT-PCR was performed on total mRNA from newborn forebrain of wild-type (grey bars) and *trkB*^-/-^*;trkC*^+/- ^(red bars) mice using primers specific for *Cebpa*, *Cebpb *and *Neurod*. After normalization to *gapdh*, mRNA levels were expressed relative to those for the wild-type animals, which were given the arbitrary value of 1. Error bars represent the standard error based on three independent experiments. *p < 0.05, **p < 0.002.

The above results are consistent with Trk receptor signaling regulating either the recruitment or the activity of C/EBP and NeuroD transcription factors to target promoters. The major Trk receptors expressed in the developing central nervous system (CNS) are TrkB and TrkC, which function in a redundant manner in promoting neuronal survival during CNS development [17]. To address the role of Trk signaling in C/EBP and NeuroD promoter recruitment we performed ChIP experiments on newborn forebrains of mice lacking TrkB and one allele of the gene encoding TrkC (*Ntrk2*^-/-^*Ntrk3*^+/- ^mice), thereby substantially lowering the level of neurotrophin/Trk signaling in the developing CNS. These experiments showed that the amount of C/EBPs and NeuroD present on the *Fos*, *Egr1 *and *Egr2 *promoters was significantly lower in forebrains where Trk signaling was reduced compared to wild-type forebrain (Figure 6a–c) or mouse forebrain lacking only TrkB (which showed an intermediate phenotype, data not shown), whereas ChIP for H3 (a control for chromatin quality) did not show any difference (Figure 6d). Importantly, expression of *Cebpa*, *Cebpb *and *Neurod*, as measured by qRT-PCR, was unaffected by the reduced Trk receptor signaling (Figure 6e).

### C/EBPα and NeuroD form a complex *in vitro *and *in vivo*

**Figure 7:**
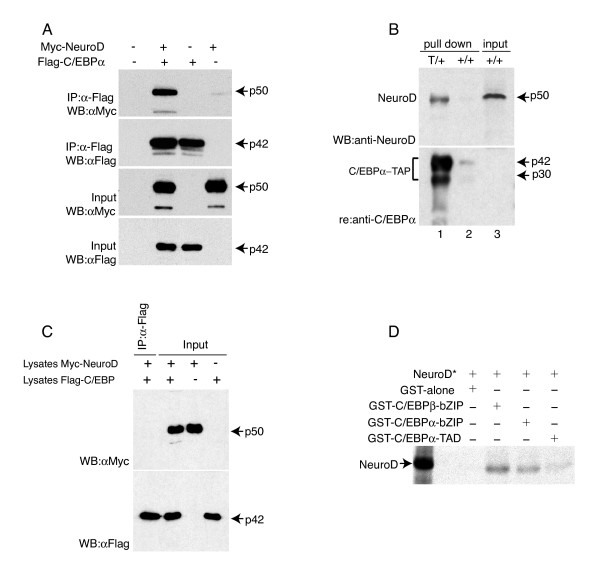
The bHLH transcription factor NeuroD co-immunoprecipitates and physically interacts with C/EBPα and β. **(a) **Co-immunoprecipitation (IP) assay and western blot analysis. Q2bn cells were transfected as indicated, and lysates were subjected to a Co-IP assay with an anti-Flag antibody and were immunoblotted (WB) with anti-Myc and anti-Flag antibodies. Direct WB analysis was used to monitor protein expression levels. **(b) **WB, using an anti-NeuroD antibody, of TEV-released IgG pull down of 5 mg nuclear extracts from E16.5 forebrain of either *Cebpa*^*TAP*/+ ^(T/+, lane 1) or +/+ (lane 2) mice; 100 μg of input lysate (+/+) was loaded in lane 3. Note the specific presence of NeuroD (50 kDa) in the T/+ pull down. The blot was reprobed with an anti-C/EBPα antibody showing the specific pull down of C/EBPα (42 kDa and 30 kDa isoforms) in the T/+ sample (lane 1). C/EBPα in 100 μg of nuclear extract was below detectable levels by WB (lane 3). **(c) **NeuroD does not associate with CEBPα upon mixing lysates from myc-NeuroD transfected cells with lysates from flag-C/EBPα transfected cells.**(d) **Direct interaction of NeuroD to C/EBPα and β. *In vitro *translated NeuroD was subjected to a pull down assay using the indicated GST fusion proteins: GST-alone, GST-C/EBPβ-bZIP. GST-C/EBPα-bZIP., GST-C/EBPα-TAD. Autoradiograph revealed that NeuroD preferentially bound the bZIP domain of C/EBPα or β. *Radiolabeled.

While the *Fos *promoter contains consensus-binding sites for both C/EBP and Ebox proteins, a canonical consensus Ebox is not present in the *Egr1 *or *Egr2 *SRE homology regions. This, along with the observed correlation between C/EBP and NeuroD promoter recruitment, suggests that the two factors are recruited as a complex through a cognate binding site for one of the two (most likely a C/EBP). To determine whether C/EBPα and NeuroD were able to associate we co-transfected Q2bn cells with expression vectors for FLAG-tagged C/EBPα and Myc-tagged NeuroD and performed co-immopreciptiation analysis. This showed that Myc-NeuroD was efficiently co-precipitated with FLAG-C/EBPα, demonstrating that the two factors are able to form a complex (Figure 7a). To address the issue of whether such a complex exists under physiological conditions in neuronal cells we used a mouse strain in which a tandem affinity purification (TAP) tag [18] has been fused to the carboxyl terminus of C/EBPα. The TAP-tag contains an immunoglobulin (Ig)-binding domain from *S. aureus *protein A, which allows tagged proteins to selectively bind to a rabbit IgG-agarose matrix. Thus, nuclear extracts from E16.5 forebrain (a time point when the forebrain is enriched in neuronal cells) of mice heterozygous for the *Cebpa *TAP allele (*Cebpa*^T/+ ^mice) and wild-type controls were subjected to IgG-agarose pull-down and retained protein was analyzed for the presence of NeuroD by western blotting. As expected, the TAP-tagged C/EBPα isoforms were present in the pull-down of *Cebpa*^T/+ ^lysates (Figure 7b, lower panel). We observed that NeuroD was also selectively retained by IgG-agarose in *Cebpa*^T/+ ^lysates (Figure 7b, upper panel), indicating a complex formation with C/EBPα *in vivo*. While these results are fully consistent with the existence of C/EBPα-NeuroD complexes in forebrain neurons, we could not rule out that such complexes formed post-lysis. We therefore transfected Q2bn cells separately with FLAG-C/EBPα and Myc-NeuroD expression vectors and mixed lysates from these cells prior to co-immunoprecipitation. This did not lead to detectable complex formation, whereas C/EBPα-NeuroD complexes were readily detectable in lysates of Q2bn cells cotransfected with the two expression vectors (Figure 7c). Finally, to address the issue of whether the C/EBPα-NeuroD interaction was direct we performed glutathione S-transferase (GST) pull-downs using C/EBPα-GST fusions of the conserved carboxy-terminal bZIP or amino-terminal transactivation domain (TAD) and *in vitro *translated NeuroD. Of these, NeuroD associated strongly with the C/EBPα bZIP and only weakly with the TAD. Consistent with the high conservation of the bZIP domain between C/EBP isoforms, a GST fusion of the C/EBPβ bZIP region was also able to pull down NeuroD (Figure 7d). Together, these results show that NeuroD can interact with C/EBPs through their conserved bZIP region. Since C/EBPα-NeuroD complexes can be isolated from both transfected cells and forebrain suggests that these molecules are co-expressed and interact in neurons.

### C/EBP depletion leads to defective cortical dendritic differentiation *in vivo*

**Figure 8:**
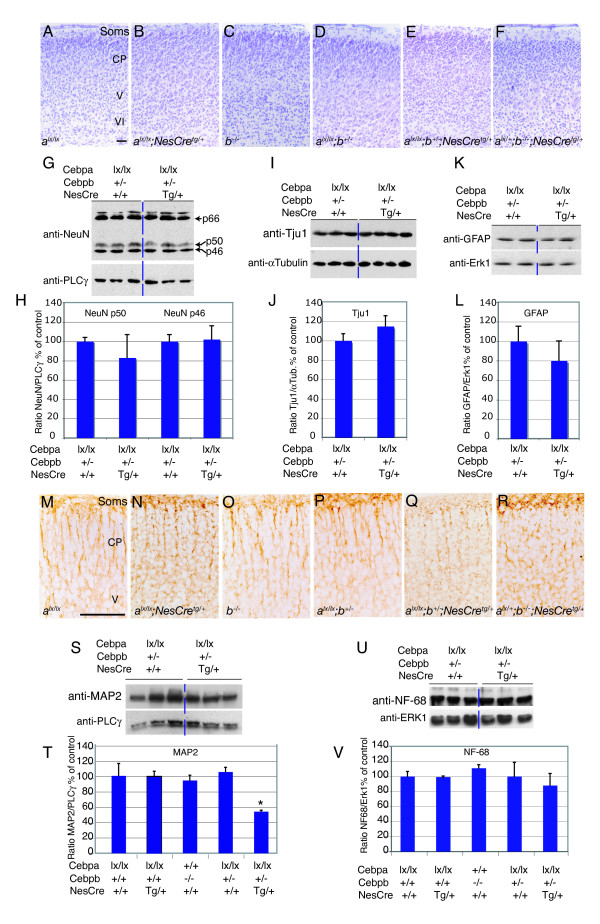
Cortical dendritic differentiation is affected in mice carrying decreasing amounts of functional *Cebpa/b *alleles. **(a-f) **Cresyl violet staining showing a normal lamination pattern in newborn mutant and control mice. **(g,i,k) **Representative western blots of NeuN, Tju1, and GFAP protein levels in P0 cortices of mutant (*Cebpa*^lx/lx^*;Cebpb*^+/-^*;NesCre*^tg/+^) and control (*Cebpa*^lx/lx^*;Cebpb*^+/-^) mice. To control for protein loading, blots were re-probed with anti-PLCγ, anti-α-tubulin, and anti-Erk1 respectively. **(h,j,l) **Densitometric analysis of NeuN, Tju1, and GFAP protein levels in P0 cortical lysates from mutant and control mice. *P *> 0.05 in all cases. **(m-r) **MAP2 immunostaining in newborn *Cebpa*^lx/lx^, *Cebpa*^lx/lx^*;NesCre*^tg/+^, *Cebpb*^-/-^, and *Cebpa*^lx/lx^*;cebp*^+/- ^mouse cortices compared to cortices with decreasing amounts of functional *Cebpa/b *alleles, *Cebpa*^lx/lx^*;Cebpb*^+/-^*;NesCre*^tg/+ ^(r), *Cebpa*^lx/+^*;Cebpb*^-/-^*;NesCre*^tg/+ ^(s). **(s,u) **Representative western blots of MAP2 and NF68 protein levels in P0 cortices of mutant (*Cebpa*^lx/lx^*;Cebpb*^+/-^*;NesCre*^tg/+^) and control (*Cebpa*^lx/lx^*;Cebpb*^+/-^) mice. To control for protein loading, blots were re-probed with anti-PLCγ and anti-Erk1 antibodies respectively. **(t) **Densitometric analysis of MAP2 protein levels in cortical lysates of control and *Cebpa/b *mutant mice (*p = 0.002, compared to control *Cebpa*^lx/*lx*^*;Cebp*^+/-^). **(v) **Densitometric analyses of NF68 protein levels in cortical lysates of controls and *Cebpa/b *mutant mice. Soms, somatosensory cortex; CP, cortical plate; V and VI, indicate cortical layers V and VI, respectively. Arrows in (h) indicate the kDa of the bands recognized by NeuN antibodies. Scale bars: 100 μm (b-g); 50 μm (n-s).

**Figure 9:**
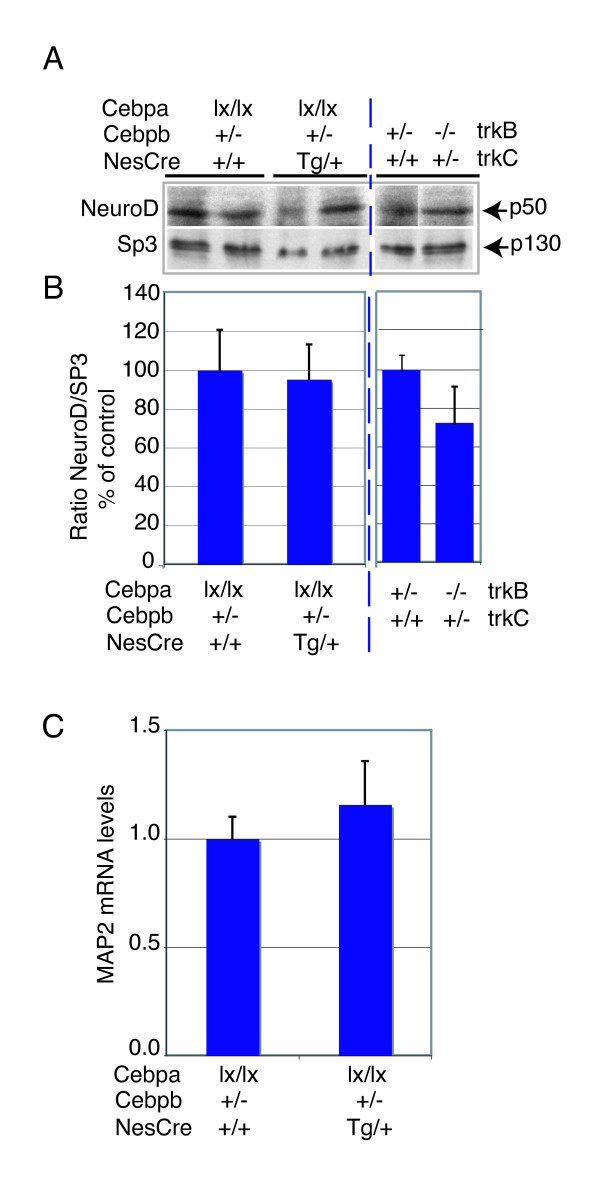
NeuroD expression is normal in *Cebpa/b *and *trkB/C *deficient brains. **(a) **NeuroD protein expression in *Cebpa/b *deficient brains or *trkB/C *deficient brains compared to controls. Blots were re-probed with anti-Sp3 antibodies to control for protein loading. *P *> 0.5 in both cases. **(b) **Densitometric analysis of NeuroD protein levels in cortical lysates of controls, *Cebpa/b *and *trkB/C *mutant mice. **(c) ***MAP2 *mRNA levels were not affected in *Cebpa*/*b *deficient brains. qRT-PCR was performed on total mRNA from newborn forebrains of control (*Cebpa*^lx/lx^*;Cebpb*^+/-^) and mutant (*Cebpa*^lx/lx^*;Cebpb*^+/-^*;NesCre*^tg/+^) mice using primers specific for *MAP2A/2B *isoforms. After normalization to *ubiquitin*, mRNA levels were expressed relative to those for the control animals, which were given the arbitrary value of 1. Presented is a representative result of two independent experiments. Standard error is for three animals of each genotype.

We next addressed the role of C/EBPs in neuronal development. Mice lacking C/EBPα die at birth [19], combined *Cebpa *and *Cebpb *null alleles are embryonic lethal, and mice carrying three *Cebpa *and *Cebpb *null alleles die at birth [16]. Therefore, to facilitate the analysis we used conditional inactivation of the *Cebpa *gene using a floxed *Cebpa *allele (*Cebpa*^lx ^allele [20]) and a Nestin-Cre transgene (NesCre [5]), and mice homozygous for the *Cebpb *null allele. Southern blot analysis showed that NesCre recombination of the *Cebpa*^lx ^allele was virtually complete in the CNS at postnatal day 0 (P0; supplemental Figure 3 in Additional data file 1). Moreover, as the co-expression of *Cebpa*/*b *in neurons suggested that they could function in a redundant manner, we also combined the two mutations. In this case the double mutant (*Cebpa*^lx/lx^*;Cebpb*^-/-^;*NesCre*^tg/+^) died at birth, whereas mutants carrying up to three *Cebpa *and *Cebpb *deleted alleles (*Cebpa*^lx/lx^*;Cebpb*^-/+^;*NesCre*^tg/+^, and *Cebpa*^lx/l+^*;Cebpb*^-/-^;*NesCre*^tg/+^) were viable and used in this analysis. Histological analysis of P0 cortices showed no gross morphological defects or alteration of cortical layering upon inactivation of up to three *Cebpa *and *Cebpb *alleles (Figure 8a–f). Likewise, expression of the general neuronal markers NeuN (Figure 8g, h) and βIII-tubulin (Tju1; Figure 8i, j) was not decreased even in *Cebpa*^lx/lx^*;Cebpb*^+/-^*NesCre*^tg/+ ^cortices, nor was any difference seen in expression of the glial marker glial fibrillary acidic protein (GFAP) (Figure 8k, l). No imbalance in neuronal versus glial lineage differentiation was therefore detected. When markers specific for neuronal dendrites (microtubule-associated protein 2, MAP2) and axons (68 kDa neurofilament protein, NF68) were analyzed, MAP2 staining of cortical dendrites was not diminished upon deletion of two *Cebpa *or *Cebpb *alleles, but was severely disrupted in the absence of three alleles (Figure 8m–r). Quantification showed that loss of three alleles additionally led to a significant decrease in the amount of MAP2 present in the forebrain (Figure 8s, t); no difference in axonal NF68 staining (data not shown) or total amount of NF68 (Figure 8u, v) was observed, indicating intact axonal differentiation. Given the previously reported regulation of MAP2 by NeuroD [21], NeuroD protein expression in *Cebpa/b *deficient brains was investigated and found to be normal (Figure 9a, b). We also investigated whether MAP2 expression was down-regulated upon loss of *Cebpa/b*; this was found not to be the case, as MAP2 expression at the mRNA level was not affected (Figure 9c). We therefore propose that the observed reduction in MAP2 protein is due to loss of MAP2-containing dendritic structures. Another possibility could be defective mRNA localization and translation, leading to reduced accumulation of MAP2 protein.

**Figure 10:**
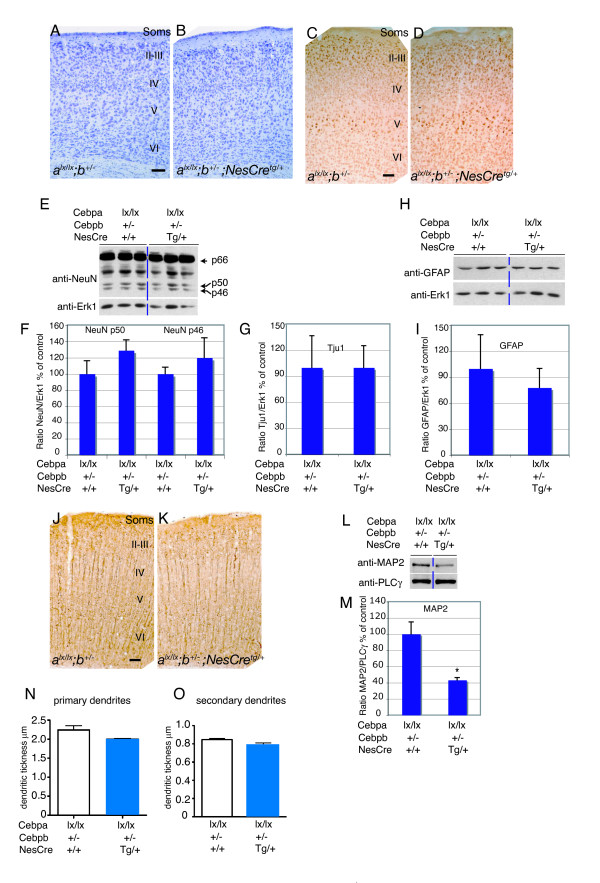
Neocortical development in mice with compound *Cebpa/b *null alleles. Coronal sections of adult (12 weeks old) cortices of control (*Cebpa*^lx/lx^*;cebp*^+/-^) and mutant (*Cebpa*^lx/lx^*;Cebpb*^+/-^*;NesCre*^tg/+^) mice were stained with **(a,b) **Cresyl violet, or immunostained for **(c,d) **NeuN. **(e,h) **Representative western blots for NeuN and GFAP protein levels in adult cortical lysates from control and mutant mice. To control for protein loading, blots were re-probed with an anti-Erk1 antibody. **(f,g,i) **Densitometric analysis of NeuN, Tju1, and GFAP protein levels in adult cortical lysates of control and mutant mice. **(j,k) **Immunostaining for MAP2 in control and mutant mice. **(l) **Representative western blots of MAP2 protein levels in adult cortices of mutant (*Cebpa*^lx/lx^*;Cebpb*^+/-^*;NesCre*^tg/+^) and control (*Cebpa*^lx/lx^*;Cebpb*^+/-^) mice. To control for protein loading, blots were re-probed with anti-PLCγ antibodies. **(m) **Densitometric analysis of MAP2 protein levels reveals significant decrease in mutant compared to control mice. *P = 0.019. **(n,o) **Mean thickness of primary and secondary dendrites of pyramidal neurons located in the primary somatosensory cortex (visualized with the Golgi method). II to VI indicate the different cortical layers. Scale bars (a-d,j,k): 100 μm.

The possibility remained that the differences observed in dendritic differentiation reflected a developmental delay, rather than a defect. We therefore performed similar analyses with 12-week-old mice. Also in this case, cortical layering (as determined by cresyl violet staining; Figure 10a, b) and neuronal numbers (assayed by NeuN immunostaining; Figure 10c, d) were unaffected. Immunoblotting confirmed equivalent levels of NeuN (Figure 10e, f), βIII-tubulin (Figure 10g) and the glial marker GFAP (Figure 10h, i), whereas the defect in MAP2 staining in *Cebpa*^lx/lx^*;Cebpb*^+/-^*;NesCre*^tg/+ ^mice (compared to *Cebpa*^lx/lx^*;Cebpb*^+/- ^controls) was still persistent (Figure 10j, k). Immunoblotting confirmed the decrease in MAP2 protein levels (Figure 10l, m). Together, these data indicate a role for C/EBPα/β in cortical dendritic differentiation. To further support this conclusion we measured the thickness of apical primary and secondary dendrites of pyramidal neurons located in the primary somatosensory cortex. The mean thickness of apical primary dendrites in *Cebpa*^lx/lx^*;Cebpb*^+/-^*;NesCre*^tg/+^mice was found to be reduced by 10% compared to *Cebpa*^lx/lx^*;Cebpb*^+/- ^controls (2.00 ± 0.02 μm and 2.24 ± 0.115 μm, respectively; Figure 10m). Similarly, the secondary dendrites were 7% thinner in mutants compared to control mice (0.79 ± 0.02 μm and 0.85 ± 0.013 μm, respectively; Figure 10n). Although the differences observed in both cases are not statically significant (p > 0.05) by ANOVA analysis, they are consistent with C/EBPs being involved in dendritic differentiation function.

## Discussion

The identification of transcriptional targets for signaling by neurotrophin receptors is fundamental to understanding the effects of neurotrophins on neuronal gene expression, differentiation and function. We here provide evidence that, in neuronal cells, C/EBPα and -β form a complex with NeuroD, and that these factors are recruited to the BDNF responsive *Fos*, *Egr1 *and *Egr2 *promoters in a manner dependent on Trk receptor signaling *in vivo*. Reducing the levels of C/EBPα and -β led to reduced Fos, Egr1 and Egr2 expression in response to BDNF in primary neuronal cultures, and to impaired terminal dendritic differentiation revealed by decreased levels of MAP2 in cortical dendrites *in vivo*. Together, these results identify NeuroD-C/EBP complexes as mediators of Trk signaling, and identify the IE genes *Fos*, *Egr1 *and *Egr2 *as downstream targets for this pathway.

### Nuclear mediators of neurotrophin signaling

Neurotrophins have multiple functions during CNS development, and mediate neuronal survival, migration, differentiation and function. These processes require activation of specific downstream targets. For example, the PI3 kinase-AKT-Foxo pathway is important for neuronal survival downstream of growth factor-induced signaling [22], and both PI3 kinase-AKT and MAPK pathways are involved in similar functions downstream of TrkB receptor-induced signaling *in vivo *[15,23] whereas the phospholipase Cγ (PLCγ)-CamK-CREB pathway regulates hippocampal plasticity downstream of TrkB [3]. *Cebpb *has been previously shown to be a neuronal transcriptional target downstream of the nerve growth factor (NGF) receptor (TrkA) in PC12 cells [24]. Evidence exists indicating that NeuroD is responsive to neurotrophin signaling *in vivo*. NeuroD is highly expressed in different tissues, including the developing neurons of the peripheral nervous system (PNS) and CNS, reaching its highest expression during differentiation in the cerebral cortex [25]. In the PNS, NeuroD is required for survival and differentiation of inner ear sensory neurons during later stages [26,27]. The loss of these sensory neurons in the vestibular-cochlear ganglia (VCG) of *neuroD *null mice resembles the phenotypes found in mice lacking the receptors TrkB or TrkC (reviewed in [28]). Moreover, the expression of *trkB *or *trkC *in the VCG of *neuroD *null mice was significantly reduced, [26], supporting a link between NeuroD and TrkB/C in the survival of newly differentiating inner ear sensory neurons. Similarly, dentate granule neurons of the CNS fail to mature in *neuroD/nex1 *double mutant mice, indicating that these molecules are critical not only for the survival of early differentiating neurons, but also for their terminal differentiation [29,30]. Although no such phenotype was observed in the neocortex of *neuroD/nex1 *animals, this may be explained by NeuroD family members being functionally redundant.

We have identified NeuroD-C/EBP complexes as novel mediators of Trk signaling *in vivo*. The finding that inhibition of these complexes (through deletion of *Cebpa *and *Cebpb *alleles) has effects on cortical differentiation similar to those seen by reduction of Trk signaling during development (through disruption of *Ntrk2 *and *Ntrk3 *alleles; supplemental Figure 4 in Additional data file 1, and previous work in which we have shown that TrkB/C cooperate in promoting the survival of differentiating hippocampal and cerebellar granule neurons [17], and that TrkB regulates functions throughout the formation of the cerebral cortex, including dendrite neuronal differentiation [5]) is, furthermore, consistent with Trk signaling through NeuroD-C/EBP being required for MAP2 induction and dendrite growth. Given that C/EBPs and NeuroD are induced during neuronal lineage commitment *in vitro*, it is interesting to speculate that their appearance allows Trk receptor signaling to regulate genes involved in subsequent terminal neuronal differentiation and function.

Of the targets found to be affected in this study, Fos and Egr1 have both been implicated in dendrite formation in PC12 cells [31,32]. Genetic ablation of *Fos*, *Egr1 *or *Egr2 *has not been reported to affect dendrite differentiation *in vivo*, suggesting that the combined downregulation of multiple targets leads to the observed effects on neuronal dendrite differentiation. It is, however, equally possible that some of the NeuroD-C/EBP targets are involved in neuronal function, since *Egr1 *has been reported to be required in the CNS for long-term synaptic plasticity (LTP) in the dentate gyrus [33]. The known functions of the affected genes are, therefore, consistent with the observed differentiation phenotype, and suggest a role for C/EBPs in the generation of LTP. However, given the possibility of functional redundancies, further genetic analysis will be required to resolve this issue.

We did not observe any effects of C/EBP depletion on gross CNS morphology or the distribution or number of neurons and glial cells, as determined by histological analysis and quantification of NeuN and GFAP. Previous reports [13,14] have shown that high levels of dominant-negative C/EBP were able to inhibit neurogenesis. Our genetic data do not, at this point, confirm a role for C/EBPs in neuronal-glial lineage decisions. However, in addition to C/EBPα and β, C/EBPδ and ε are also expressed in the CNS and may be able to compensate for loss of the α and β isoforms. In addition, due to the early embryonic lethality of mice lacking all *Cebpa *and *Cebpb *alleles, one wild-type allele was intact in our analysis, which may be sufficient for any commitment step. It should be noted that the use of high levels of dominant negative C/EBPs may affect other related transcription factors (such as C/ATF and CREB/CREM), and confirmation of the proposed C/EBP function in neuronal lineage commitment by genetic means will still be important to finally resolve this issue.

### Recruitment of C/EBP-NeuroD complexes to target promoters

The promoter elements in the *Fos*, *Egr1 *and *Egr2 *promoters that recruit C/EBPα/β and NeuroD have significant homology, but in the case of *Egr1 *and *Egr2 *do not contain a consensus Ebox. This raises the possibility that the C/EBP-NeuroD complexes that form *in vivo *in neuronal cells expressing these factors (as determined by TAP-mediated pull down from mouse forebrain tissue) are recruited through a single binding site for C/EBP, with little or no interaction between NeuroD and DNA. This notion is supported by the observation that mutation of the *Fos *promoter Ebox had no effect on *Fos *promoter activity in transient transfection assays, whereas simultaneous mutation of both the C/EBP site and the Ebox significantly decreased promoter induction by BDNF.

The recruitment of NeuroD and C/EBPα/β to IE gene promoters was impaired *in vivo *when the levels of Trk signaling were decreased by inactivation of *Ntrk2*/*Ntrk3*. This result demonstrates that Trk-mediated signaling regulates the association of these factors with chromatin, and the observation that NeuroD and C/EBP recruitment were highly correlated is consistent with these factors being recruited as a complex, as discussed above. Importantly, lowering the level of C/EBP through *Cebpa*/*Cebpb *inactivation decreased the inducibility of all three IE gene promoters in response to BDNF, providing a direct link between the level of C/EBP (and by inference C/EBP-NeuroD complexes) available and neurotrophin-mediated gene activation. While C/EBPs, therefore, are an essential component of TrkB/TrkC mediated IE gene activation, the events linking Trk signaling to C/EBP and/or NeuroD remain to be fully understood at this point. Both C/EBPs and NeuroD are known to be phosphorylated in response to extracellular signaling; however, the physiological relevance of these phosphorylation events are yet to be addressed genetically. Perhaps most relevantly, from *in vitro *studies, phosphorylation of C/EBPβ Thr188 by ERK, and Thr217 by ribosomal S6 kinase (Rsk), both of which are kinases downstream of MEK [34], have been proposed to be required for their role in neuronal lineage commitment [13,14]. We find that Trk-mediated IE gene induction depends on the Shc adaptor-binding site, a principal function of which is to mediate high-level sustained ERK activation in cultured neurons [15]. It is therefore possible that, also in the context of a NeuroD-C/EBP complex, this pathway regulates C/EBP activity. Experiments to address this question genetically are currently underway.

In summary, we have shown here that C/EBPα and NeuroD form complexes *in vivo *and *in vit*ro. These may define a novel class of C/EBP-bHLH transcription factor complexes of widespread importance, as we have recently shown that a similar interaction occurs between C/EBPα and SREBP-1 in hepatocytes [35]. ChIP analysis and genetic depletion demonstrated that recruitment of C/EBP and NeuroD to IE gene promoter chromatin occurred concurrently in a Trk-dependent manner, and was required for IE gene induction by neurotrophins. The physiological result of C/EBP depletion was reduced MAP2 expression and impaired cortical dendritic differentiation. We have, therefore, identified C/EBP-NeuroD complexes as novel mediators of Trk signaling, and provide evidence that such complexes function in late stages of neuronal development to promote terminal neuronal differentiation.

## Materials and methods

### RNA preparation for microarray analysis and RT-PCR

RNA was isolated from E15.5 cortical neurons of +/+ and *trkB*^*SHC*/*SHC *^mice with TRIzol according to the protocol supplied by the manufacturer (GIBCO Life Technologies (now Invitrogen), Milan, Italy), and labeled according to the one-cycle eukaryotic target labeling protocol from Affymetrix (Santa Clara, CA, USA). The samples were hybridized to Affymetrix Murine Genome U74Av2 GENEChips. For RT-PCR or qPCR, cDNA was prepared from total RNA using random hexamer primers and MuLV reverse transcriptase (Roche, Milan, Italy) or Ready-to-go T-primed First Strand Kit (Amersham Biosciences, Milan, Italy) as described by the manufacturers. The sequences of the primers used for the different PCR reactions are listed in Table 1.

**Table 1:** Primer sequences used for RT-PCR, q PCR and ChIP

	Gene	Sense	Antisense
RT-PCR	*Cebpa*	aaggccaagaagtcggtgga	cagttcacggctcagctgtt
	*Cebpb*	gcgcgagcgcaacaacatc	tgcttgaacaagttccgcag
	*Neurod*	tcaaccctcggactttcttg	gcagtcagttagggggcttt
	*Ubiquitin*	tggctattaattattcggtctgcat	gcaagtggctagagtgcagagtaa
qPCR	*Egr1*	gttatcccagccaaacgactc	ggttcaggccacaaagtgtt
	*Fos*	cccatccttacggactccc	gagatagctgctctactttgcc
	*Egr2*	ggaccacctctactctccg	tgggatcataggaatgagacctg
	*Cebpa*	gtcactggtcaactccagcac	caagaacagcaacgagtaccg
	*Cebpb*	ggagacgcagcacaaggt	agctgcttgaacaagttccg
	*Neurod*	atgaccaaatcatacagcgagag	tctgcctcgtgttcctcgt
	*MAP2A/B*	aaagttgcctccagttccattt	tctttgattccgtgggcattt
	*gapdh*	aactttggcattgtggaagg	acacattgggggtaggaaca
ChIP	Promoter *Egr1*	tgcccaccactcttggatgg	ttcaagggtctggaacagca
	CD *Egr1*	gttatcccagccaaacgactc	ggttcaggccacaaagtgtt
	Promoter *Fos*	gctcgccttctctgcctttc	gcgctctgtcgtcaactcta
	Promoter Fos*	tccctccctcctttacacag	cccgtcttggcatacatctt
	3' UTR *Fos*	ggctacactgtgagatccta	acaactgccagggccattag
	Promoter *Egr2*	gccctgttcctcagtccata	gccaggagttgctggtgtag
	CD *Egr2*	ggaccacctctactctccg	tgggatcataggaatgagacctg

For the ChIP experiments the same primers were used for both PCR and qPCR except where noted. *Additional primers used for *Fos *qPCR after ChIP. CD, coding region; UTR, untranslated region.

### DNA constructs

The constructs pMSRF/cebp, pMcebp/ebox, and pMebox were created as previously described [36]. Mutations in the pMSRF/cebp and pMcebp/ebox constructs were introduced as described [37], whereas for the pMebox construct the QuickChange Site-directed Mutagenesis Kit was used (Stratagene, Milan, Italy). The oligonucleotide primers used to generate pMSRF/cebp, pMcebp/ebox and pMebox constructs carried the following mutations (underlined), respectively: tgtccatcggaggacatctgcgtcagcaggtttccacggcc, tgtccatattaggaacgctgcgtcagcaggtttccacggcc, and tgtccatattaggaaatctgcgtcagcaggtttccacggcc. C/EBPα was introduced as *Bam*HI-*Eco*RI fragments in pcDNAI (Invitrogen, Milan, Italy) for *in vitro *labeling [38], and in pcDNA3.1 (Invitrogen) for transfection. Mouse NeuroD full-length cDNA was obtained by RT-PCR using RNA isolated from cortical neurons and the primers *Bam*HI/5'-cgggatccgtggaaacatgacc-3' and 5'-ggaattcactgacgtgcctcta-3'/*Eco*RI.

### Cultures of cortical progenitors and postmitotic neurons

Cortical progenitors, as well as postmitotic neurons, from mouse embryos were cultured according to previously described methods [15,39]. Briefly, cortices were collected from E12-13 mouse embryos, triturated, and then plated on poly-L-lysine (at different densities depending on the final experiment). The culture medium consisted of Neurobasal medium (GIBCO BRL (now Invitrogen), Milan, Italy), 0.5 mM glutamine, penicillin-streptomycin, 1% N2 supplement (GIBCO BRL), and basic fibroblast growth factor (bFGF) (40 ng/ml; R&D Systems, Milan, Italy). After 48 hours, medium was replaced with the same medium containing 2% B27 supplement (GIBCO BRL) instead of 1% N2 supplement. Postmitotic neurons were prepared from cerebral cortices of E15.5 mouse embryos derived from crosses of wild-type or *trkB*^*SHC*/*SHC *^homozygous mice. Cortical neurons were cultured and BDNF stimulated as described [15]. Purified recombinant BDNF (Regeneron Pharmaceuticals, Inc., Tarrytown, NY, USA) was added to the culture medium at a concentration of either 5 or 50 ng/ml, as indicated.

### Immunocytochemistry

Cortical progenitors after either 1 or 4 days in vitro (DIV) were fixed in 4% paraformaldehyde (PFA) for 20 minutes. Washes were performed with 10 mM phosphate-buffered saline (PBS)/glycine to neutralize the PFA. The cells were permeabilized with 0.5% NP-40/PBS for 5 minutes. Blocking solution was added for 1 hour (10% NHS, 0.3% Carrageenan Lambda (Sigma, Milan, Italy), 0.5% TritonX100 in 50 mM TBS) at room temperature, followed by incubation with either a mouse monoclonal anti-MAP2 antibody (1:300, clone AP20, ChemiconMilan, Italy) or a mouse monoclonal anti-Nestin antibody (1:300, clone 401, Chemicon) in 1% normal horse serum (NHS), 0.3% Carrageenan Lambda, 0.5% TritonX100, 50 mM Tris-buffered saline (TBS), O/N at 4°C. Immunostainings were visualized using a fluorescein-conjugated goat anti-mouse IgG (H+L) (1:200 in TBS, Jackson IR, Milan, Italy). The slides were mounted with Vectashield mounting medium (Vector H2000, Milan, Italy).

### Transfection of cortical neurons and luciferase assay

Cortical neurons were plated at 3 × 10^6 ^cells per 60 mm well. Transfections were carried out on either 3 or 4DIV using Lipofectamine 2000 reagent (Invitrogen) according to the manufacturer's protocol. Briefly, one 60 mm well of cells was transfected with 2.3 μg of reporter plasmid plus 3 μg of the plasmid ras sarcoma virus long terminal repeat containing the Escherichia coli beta-galactosidase gene (pRSV-β-gal). Then, 24 hours after transfection, cells were left unstimulated or stimulated with 50 ng/ml of BDNF for 2 hours. Cells were then lysed in 250 μl of Triton lysis buffer (100 mM potassium phosphate pH 7.8, 0.2% Triton X-100, 1 mM dithiothreitol (DTT)). Cell debris was removed by centrifugation, and the lysates were used in luciferase and β-gal assays. The luciferase activity was measured using a Lumat LB 9507 luminometer (Berthold Technologies Wildbad, Germany). The fold induction was defined as the ratio of β-gal normalized luciferase activity in lysates of stimulated cells relative to the normalized luciferase activity from unstimulated cells.

### Co-transfection assay

Plasmids used for cotransfections included pcDNA3.1 (Invitrogen) driving the expression of C/EBPα or NeuroD, and a wild type (pWT) or mutated (pMcebp/ebox) *Fos *promoter driving expression of luciferase. NIH3T3 cells were grown to 60% to 80% confluence in 30 mm wells and transfected with a total of 1.5 μg of DNA including 250 ng of the luciferase reporter plasmid and 250 ng of pRSV-β-gal plasmid for normalization. Cells were transfected using FuGENE 6 (Roche) according to the manufacturer's protocol. After 24 hours, cells were harvested, lysed, and the β-galactosidase and luciferase activities were measured as described above.

### Co-immunoprecipitation assay and western analysis

pCMV-Tag3 vector (Stratagene, Milan, Italy) was used to express NeuroD possesssing one Myc-epitope tag, and pcDNA3.1 (Invitrogen) was used to express C/EBPα possessing one Flag-epitope tag. Q2bn cells were grown in DMEM supplemented with 8% fetal bovine serum and 2% chicken serum, transfected using calcium phosphate, and harvested 24 hours after transfection. Cells were disrupted in lysis buffer (50 mM Tris pH 7.4, 1.5 mM MgCl_2_, 100 mM NaCl, 0.5% Triton X-100) supplemented with a protease inhibitor cocktail (Roche) on ice for 15 minutes, and then incubated at room temperature for 30 minutes with 0.25 u/μl of benzonase (Sigma). After clarification by centrifugation, lysates were subjected to Co-IP using 20 μl of anti-Flag M2 affinity gel beads (Sigma) at 4°C overnight (O/N). The beads were collected and washed, and the bound proteins were eluted by a competition with 3X FLAG peptide (Sigma), and analyzed by SDS-PAGE followed by western blotting using anti-Myc tag antibody (1:2000, Ab9106, Abcam, Cambridge, UK) and anti-Flag tag antibody (1:2500, Sigma). Analysis of pERK1/2 and pAKT in stimulated cortical neurons derived from cerebral cortices of E12.5 mouse embryos of controls and different *Cebpa *and *Cebpb *mutants was carried out using western blots as described [15]. The monoclonal anti-pERK1/2 antibody was used at 1:2000 (clone E10, New England Biolabs, Frankfurt, Germany), the rabbit polyclonal anti-pAKT (Ser473) was used at 1:1000 (Cell Signaling, Frankfurt, Germany), and the monoclonal anti-α-tubulin antibody was used at 1:20.000 (clone DM1A, Sigma).

### GST pull down assay

The GST fusions C/EBPβ bZIP (amino acids 231 to 328 of chicken C/EBPβ) and C/EBPα bZIP (amino acids 256 to 358 of rat C/EBPα) were constructed by PCR amplification and cloning into pGEX4T-1 (Pharmacia, Milan, Italy). GST C/EBPα TAD (amino acids 1 to 96) has been described [40]. ^35^S-labeled NeuroD was prepared using the Promega TNT T7 system and L-^35^S-methionine (Amersham Biosciences) according to the manufacturer's suggestions. Pull down experiments were carried out by pre-incubating 100 μl of bead suspension (corresponding to about 500 ng protein) with 50 μl 10% bovine serum albumin (BSA) and 350 μl pull down buffer (50 mM Tris pH 8, 250 mM NaCl, 0.5% NP40) for 30 minutes at room temperature with gentle shaking. ^35^S-labeled protein (5 μl) was subsequently added, and the incubation was continued for 1 hour. The beads were then washed 3 times with ice-cold NETN buffer (0.1 M NaCl, 1 mM EDTA, 10 mM Tris pH 8.0, 0.5% NP40), pelleted, and resuspended in 20 μl of 2X SDS buffer (20% glycerol, 4% SDS, 10% β-mercaptoethanol, 125 mM Tris-Cl pH 6.8, 0.2% bromophenol blue). The samples were boiled for 5 minutes, and the beads were pelleted. The supernatants were resolved by SDS-PAGE (12.5%). Labeled proteins were detected on a Fuji BAS2040 phosphorimager (Milan, Italy).

### Tandem affinity purification

Wild-type (+/+) and *Cebpa*^*TAP*/+ ^(T/+) E16.5 forebrains were dissected, and then lysed in hypotonic buffer (25 mM Tris pH 7.4, 50 mM KCl, 2 mM MgCl2, 1 mM EDTA, 1 mM DTT, protease inhibitors from Sigma) for 20 minutes on ice. The samples were centrifuged at 4,000 rpm for 20 minutes at 4°C to collect the nuclei, which were washed in hypotonic buffer and resuspended in benzonase buffer (50 mM Tris pH 7.4, 1.5 mM MgCl_2_, 100 mM NaCl, 10% glycerol and 1 mM DTT and protease inhibitors). Benzonase (Novagen, Milan, Italy) was then added at 0.25 u/μl and left at room temperature for 25 minutes, after which lysates were supplemented with 0.2% NP40 and left on ice for 10 minutes. The nuclear extracts were collected by centrifugation at 14,000 rpm at 4°C for 20 minutes, and 5 mg of this were used in IP after a pre-clearing step with proteinA sepharose beads (Pharmacia) at 4°C for 30 minutes. We added 20 μl rabbit IgG agarose beads (Sigma) to the extracts and incubated them with gentle rocking at 4°C for 2 hours. The beads were washed three times with lysis buffer followed by TEV protease buffer (10 mM Hepes-KOH pH 8.0, 150 mM NaCl, 0.1% NP-40, 0.5 mM EDTA, 1 mM DTT). TEV protease (Invitrogen) was then added at 0.3 u/μl and left overnight at 4°C. Proteins in the supernatant of centrifuged samples were precipitated using acetone, dissolved in 20 μl of sample buffer, and loaded on a 10% SDS-PAGE gel. Western blot analysis was performed using an anti-NeuroD antibody (1:500; D-20, SantaCruz, Milan, Italy) in 5% BSA. Stripped membranes were re-probed with an anti-C/EBPα antibody (1:500; 14AA, SantaCruz).

### ChIP analysis

ChIP assays were performed essentially as previously described [41]. E16.5 or P0 wild-type mouse forebrains were quickly dissected, cut into small pieces, and incubated for 10 minutes in 1% PFA at 37°C. The tissues were collected, washed in PBS, and resuspended in lysis buffer (1% SDS, 10 mM EDTA, 50 mM Tris-HCl pH 8.1) containing 1 mM phenylmethylsulphonyl fluoride (PMSF) and 2 mg/ml aprotinin. After sonication, lysates were cleared by centrifugation and diluted 10-fold with dilution buffer (0.01% SDS, 1% Triton 100X, 1.2 mM EDTA, 16.7 mM Tris-HCl pH 8.1, 150 mM NaCl). After a preclearing step using a salmon sperm DNA/ProteinA agarose slurry (50% slurry, Upstate, Milan, Italy), the chromatin was incubated O/N at 4°C with either rabbit Ig, or 2 μl of C/EBPα (14AA, SantaCruz), C/EBPβ (#3082 polyclonal, Cell Signaling), NeuroD (Santa Cruz), histone H3 (Abcam), or acetylated histone H3 (Upstate) antibodies. IP complexes were collected using protein A/G sepharose beads, and washed twice with dilution buffer, once with 500 mM NaCl, and once with Tris-EDTA buffer. After the final wash, 250 μl of elution buffer (1% SDS, 0.1 M NaHCO_3_) was added, and the beads were rotated at room temperature for 15 minutes. The elute was supplemented with 5 M NaCl and incubated for 4 hours at 65°C to reverse the formaldehyde cross-linking. To analyze the DNA, samples were treated with 40 ng/μl of proteinase K (Merck, Milan, Italy) for 1 hour at 45°C, extracted with phenol:chloroform, and ethanol-precipitated in the presence of glycogen as carrier. The final IP DNA products were resuspended in 20 μl of 10 mM Tris-HCl and 1 mM EDTA, of which 2 μl was used for PCR analysis with 50 pmol of each primer (Table 1) in a final volume of 50 μl.

### Quantitative PCR

qPCR or qRT-PCR was performed using the DyNAmoSYBR Green qPCR kit according to the manufacturer's protocol (Finnzymes, Milan, Italy) using the primers indicated in Table 1. For the ChIP, serial dilutions of IP DNA were subjected to real time PCR. After running the qPCR reactions, crossing threshold (T_c_) values were determined for each sample. The T_c _value was defined as the cycle at which the fluorescence rises above a baseline threshold, using iCycler software (MJ Research, Cambridge, MA). To normalize for variations in the amount of starting DNA, a ΔT_c _value was calculated by subtracting the T_c _value for the immunoprecipitated sample from the T_c _value for the corresponding input DNA. DNA quantities were then expressed as percentages of corresponding input using the ChIP sample as a percentage of input = 2^Δ(Tc) ^× 100. Only when IP samples contained > 1.5 times as much DNA as the Ig samples were they considered to have sufficient DNA for analysis [42].

### Transgenic mouse strains

The methods used to generate the knockouts of *trkB*, *trkC*, and *trkB*^*SHC *^have been previously described [15,17], as well as those used to generate the knockouts of *Cebpa *(generously provided by Drs D Tenen (Harvard University, Boston MA, USA) and G Darlington (Baylor College of Medicine, Houston TX, USA) and *Cebpb *(generously provided by Drs PF Johnson and E Sterneck (National Cancer Institute, Bethesda DC, USA), the floxed *Cebpa (*generously provided by Dr YH Lee, Institute of Molecular Biology, Accademia Sinica, Taipei, Taiwan), and the transgenic NestinCre mouse lines [5,19,20,43]. The C/EBPα-C-TAP strain was generated according to [18] (O Ermakova and C Nerlov, unpublished data).

### Histology and protein expression levels

Histological and immunohistochemical (IHC) analyses were carried out as described previously [3]. Brain tissue biochemistry was performed as described [5]. The specific antibodies used to evaluate neuronal differentiation were NeuN (1:500 WB, 1:100 IHC, clone A60, Chemicon), βIII-tubulin (1:10,000, clone Tuj.1, AbCam), GFAP (H-50; 1:500, polyclonal, Santa Cruz), MAP2 (1:300, clone AP20, Chemicon) and NF68 (AB1983; 1:250, polyclonal, Chemicon). The antibodies anti-PLCγ-1 (1:2,000, mixed monoclonal, Upstate), anti-Erk1 (1:3,000, monoclonal, Zymed, Milan, Italy), and anti-α-tubulin (1:20,000, clone DM1A, Sigma) were used to control for protein loading. For the analysis of NeuroD protein expression, P0 cortices from *Cebpa/b *and *trkB/C *mutants and controls were lysed in (50 mM Tris pH 7.4, 1.5 mM MgCl_2_, 100 mM NaCl, 0.5% Triton X-100) supplemented with a protease inhibitor cocktail (Roche) on ice for 15 minutes, and then incubated at room temperature for 30 minutes with 0.25 u/μl of benzonase (Sigma). Western blot analysis was performed using an anti-NeuroD antibody (1:500, D-20, SantaCruz) in 5% BSA. Stripped membranes were re-probed with an anti-SP3 antibody (D-20X; 1:1,000, polyclonal, SantaCruz). Densitometric analysis [44] of imaged blots was used to compare the expression levels of proteins in mutant and control brains, and was performed on three different blots for each antibody. The ratios obtained for mutant and control samples were compared statistically using Student's *t*-test.

### Golgi method and analysis of dendritic thickness

Mice were perfused with 4% PFA in phosphate buffer. Brains were dissected and post-fixed in 4% PFA. Golgi staining was performed according to the Golgi-Kopsch method, as described previously [45]. Briefly, blocks of brain tissue (approximately 2.5 mm thickness) were prepared and soaked in a solution containing 2.5% potassium-dichromate for 1 day at room temperature in the dark. The solution was then replaced with fresh of 2.5% potassium-dichromate solution, and tissues were incubated for another 6 days. Tissues were then washed and transferred to a solution containing 0.75% AgNO_3_. After 5 to 6 days, brain pieces were removed, washed in 40% and 20% ethanol, and cut into 80 μm coronal sections using a vibratom (Leica, Heidelberg, Germany). Sections were mounted on gelatin-coated slides and coverslipped with Merkoglas (Merk, Darmstadt, Germany). Analysis of dendritic thickness was performed on Golgi-impregnated sections that were uniformly dark throughout the section. Only dendrites that displayed no breaks in their staining, and were not masked by other neurons or artifacts, were evaluated. Quantitative three-dimensional analyses were performed using a combined hardware-software system (NeuroLucida, Microbrightfields Inc., Colchester, VT, USA) controlling the x-y-z axis of the microscope (Axioscop Imaging, Zeiss, Rehlingen, Germany), and a microscope-mounted video camera (Hitachi, Tokyo, Japan). The three-dimensional reconstruction was done using a 100× objective (NA: 1.4; oil immersion) and the NeuroLucida system. Mean dendritic thickness was calculated from the reconstructed dendrites with the help of NeuroExplorer (Microbrightfields Inc.). The mean, standard deviation (SD), and standard error of the mean (SEM) were calculated. ANOVA followed by a Newman-Keuls test were used for statistical analysis, and were performed using Prism 4.0 (Graph Pad, San Diego, USA).

### Authorization for the use of experimental animals

The EMBL-Monterotondo Animal Ethics Committee approved all animal procedures. We further confirm that all animal experiments conformed to Italian and European regulatory standards.

## Competing interests

The author(s) declare that they have no competing interests.

## Authors' contributions

AMC participated in the design of the study and performed the molecular biology, biochemical and genetic experiments, and the statistical analysis. CN was involved in revising the manuscript critically, and contributed to the molecular biology experiments. RGL contributed to the molecular genetics experiments. CS contributed to the biochemical and molecular genetics experiments, and participated in the sequence alignment. OBH performed the Golgi method and analysis of dendritic thickness. OB has contributed to the *in vitro *biochemical analysis of the C/EBP-NeuroD complex. LM conceived the study, participated in its design and coordination, and drafted the manuscript. All authors have read and approved the final manuscript.

## Supplementary Material

Additional file 1 Four supplementary figures. Click here for file

Additional file 2 Additional Materials and methods. Click here for file
