# Supplementary material for: Neurotrophin/Trk receptor signaling mediates C/EBPα, -β and NeuroD recruitment to immediate-early gene promoters in neuronal cells and requires C/EBPs to induce immediate-early gene transcription
Source: Neural Dev. 2007 Jan 25;2:4. doi: 10.1186/1749-8104-2-4 (PMC1796876; doi:10.1186/1749-8104-2-4)
Supplement: Additional file 1 — Four supplementary figures. [file 1749-8104-2-4-S1.pdf]

Additional Figure 1

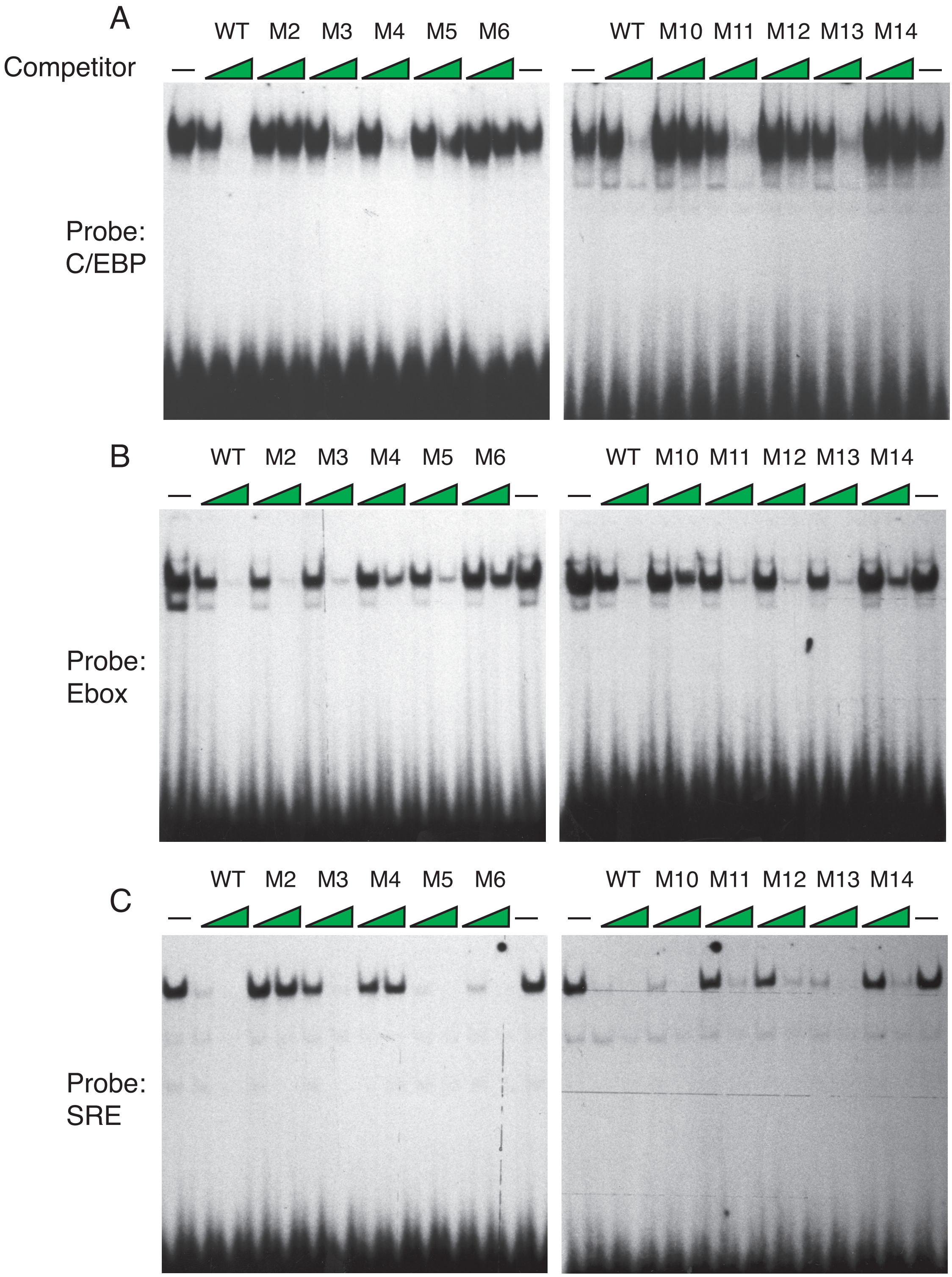

Binding properties of SRF, CEBP and bHLH proteins to their putative sites in either the WT or mutant Fos promoter-reporter constructs. (A-C) Analysis of binding to C/EBP, E-box and SRE was performed using 10µg of HeLa nuclear extract as described [2] Additional file 2. For each of the 3 probes containing only one of the 3 binding sequences (+ 1-4 random flanking nucleotides to stabilize the duplex), wild type (WT) and mutant competitors were tested at two different concentrations (50 and 500pmoles/20µl reaction, increasing left to right). Loss of competition relative to that observed with the WT competitor was used to identify mutants with loss of binding of one or more factors. For sequences of probes and competitors see Additional file 2. –, indicates no added competitor.

Additional Figure 2

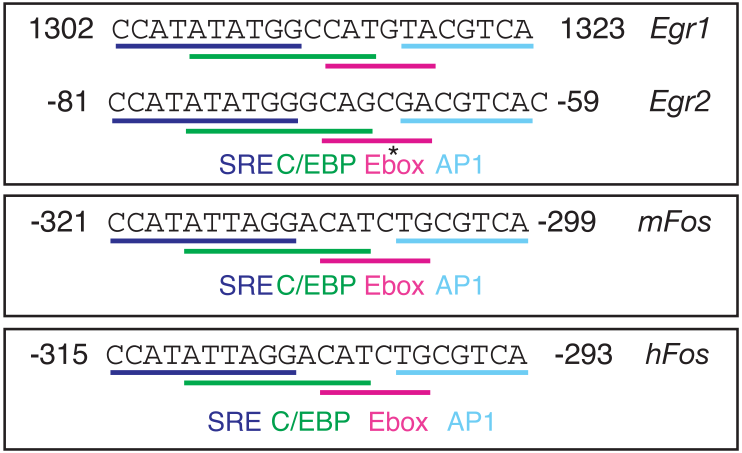

Sequence comparison between Fos, Egr1 and Egr2 proximal promoter regions. Note the similar organization of the different cis-acting elements in the proximal region of the three genes. The conserved elements are indicated with different colours. Purple for the SRE, green for the C/EBP binding site, pink for the Ebox, blue for the AP1 site. *, indicates non canonical consensus Ebox element. Comparison between the mouse and human Fos proximal promoter region is also shown.

Additional Figure 3

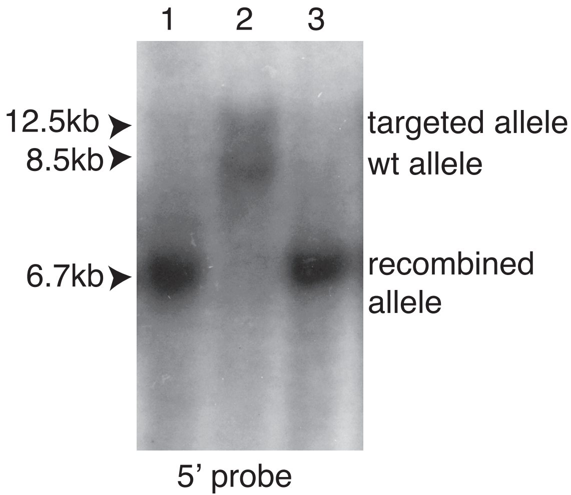

Southern blot analysis of targeted Cebpa locus performed on newborn mouse forebrains. DNA was digested with BamH1, and probed with 1.2kb 5' probe.

Additional Figure 4

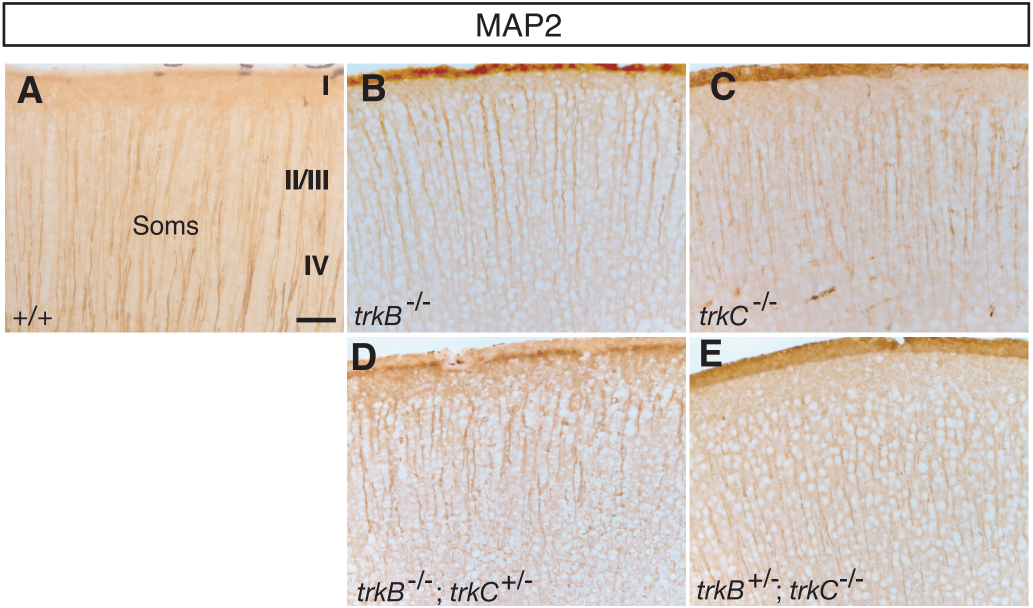

Trk-mediated cortical differentiation . (A-E) Somatosensory cortices of postnatal day 12 (P12) mice immunostained with antibodies against MAP2 reveal that mice carrying a single deletion for either trkB or trkC (Ntrk2 and Ntrk3 respectively) already show impairment in cortical dendritic differentiation (B-C, compared to A), an effect that is more pronounced upon decreasing the amount of functional alleles of either gene (D-E). Soms, somatosensory cortex. I, II/III, and IV, indicate cortical layers. Scale bar for A-E, 50μm.
