# Supplementary material for: Neurotrophin/Trk receptor signaling mediates C/EBPα, -β and NeuroD recruitment to immediate-early gene promoters in neuronal cells and requires C/EBPs to induce immediate-early gene transcription
Source: Neural Dev. 2007 Jan 25;2:4. doi: 10.1186/1749-8104-2-4 (PMC1796876; doi:10.1186/1749-8104-2-4)
Supplement: Additional file 2 — Additional Materials and methods. [file 1749-8104-2-4-S2.pdf]

Additional materials and methods

EMSA

Nuclear extracts from HeLa cells were prepared according to standard procedure [1], and 10μg were used for gel shift assays.

Oligonucleotides corresponding to the SRE, C/EBP, or Ebox, of the mouse *c-fos* promoter were end-labelled with γ^{32}P ATP using T4 polynucleotide kinase. EMSA was carried out as described [2]. For the competition experiments, excess unlabelled oligonucleotide (10-100 pmoles), corresponding to the combined SRE-C/EBP-Ebox motif, with the indicated mutations, was included in the binding reaction to determine their effects on SRF, C/EBP and Ebox binding. Sequences of probes and competitors are shown below, in all cases only one of the strands in shown. For the competitors only nucleotides differing from the WT sequence are indicated directly below the replaced nucleotide(s).

<u>Probes:</u>

```
C/EBP:    5'-  tcgaATATTAGGACATCTtgac
SRE:      5'-  gGTCCATATTAGGACt
E-box:    5'-  gAGGACATCTGCGTCc
```

<u>Competitors:</u>

```
WT        5'-  TGTCCATATTAGGACATCTGCGTCA

M2                    CGG
M3                       C
M4                        TT
M5                          G
M6                           ACG
M10                      C  G
M11                   C
M12                    G
M13                            G
M14                   TAA

WT        5'-  TGTCCATATTAGGACATCTGCGTCA
```

Promoter sequence comparison

Sequence comparison analysis of *Egr1*, *Egr2* and *Fos* promoters was based on the NCBI Accession Numbers NM_007913, NM_010118, and NM_010234. The sequences of mouse *Egr1*, *Egr2* and *Fos* were aligned using Dragon Gene Start Finder (DGSF) software, and considering significant a cut off value of 0.8 for the different binding sites.

Additional references

1. Dignam JD, Lebovitz RM, Roeder RG: **Accurate transcription initiation by RNA polymerase II in a soluble extract from isolated mammalian nuclei.** *Nucleic Acids Res* 1983, **11:** 1475-1489.
2. Nerlov C, Rorth P, Blasi F, Johnsen M: **Essential AP-1 and PEA3 binding elements in the human urokinase enhancer display cell type-specific activity.** *Oncogene* 1991, **6:** 1583-1592.
